# TIPdb: A Database of Anticancer, Antiplatelet, and Antituberculosis Phytochemicals from Indigenous Plants in Taiwan

**DOI:** 10.1155/2013/736386

**Published:** 2013-05-12

**Authors:** Ying-Chi Lin, Chia-Chi Wang, Ih-Sheng Chen, Jhao-Liang Jheng, Jih-Heng Li, Chun-Wei Tung

**Affiliations:** ^1^School of Pharmacy, College of Pharmacy, Kaohsiung Medical University, 100 Shih-Chuan 1st Road, Kaohsiung 80708, Taiwan; ^2^Ph.D. Program in Toxicology, College of Pharmacy, Kaohsiung Medical University, 100 Shih-Chuan 1st Road, Kaohsiung 80708, Taiwan

## Abstract

The unique geographic features of Taiwan are attributed to the rich indigenous and endemic plant species in Taiwan. These plants serve as resourceful bank for biologically active phytochemicals. Given that these plant-derived chemicals are prototypes of potential drugs for diseases, databases connecting the chemical structures and pharmacological activities may facilitate drug development. To enhance the utility of the data, it is desirable to develop a database of chemical compounds and corresponding activities from indigenous plants in Taiwan. A database of anticancer, antiplatelet, and antituberculosis phytochemicals from indigenous plants in Taiwan was constructed. The database, TIPdb, is composed of a standardized format of published anticancer, antiplatelet, and antituberculosis phytochemicals from indigenous plants in Taiwan. A browse function was implemented for users to browse the database in a taxonomy-based manner. Search functions can be utilized to filter records of interest by botanical name, part, chemical class, or compound name. The structured and searchable database TIPdb was constructed to serve as a comprehensive and standardized resource for anticancer, antiplatelet, and antituberculosis compounds search. The manually curated chemical structures and activities provide a great opportunity to develop quantitative structure-activity relationship models for the high-throughput screening of potential anticancer, antiplatelet, and antituberculosis drugs.

## 1. Introduction

Plants have been valuable resources of traditional remedies since ancient times and continue to be major sources and inspirations for the development of therapeutic agents [1]. It was estimated that current global market for plant-derived drugs is worth more than 20 billion and the market continues growing [2]. Many clinically important drugs, from the oldest drugs on the market to recent approved drugs, are originated from plants. As an example, anti-inflammatory and antiplatelet drug aspirin, one of the most widely prescribed drugs on the market, is a plant-derived compound originally from willow and other salicylate-rich plants. Clinically important anticancer agents, such as paciltaxel, camptothecin, and vinblastine, and many promising anticancer agents currently under clinical trials are also plant-derived compounds [3, 4]. Yet, these clinically important drugs are only from 10–15% of plant species that have been explored for pharmaceutical purpose [2]. Taiwan, including the island of Taiwan and adjacent islets, is located at the boundary of tropical and subtropical areas, with the Tropic of Cancer passing through the middle. The island of Taiwan is mountainous, with a broad range of altitude, from sea level to the highest altitude of 3900 km. Owing to the unique geographical features and location, Taiwan is rich in diversity of plants [5]. The isolation of the islands from continent further contributes to the abundance of endemic species in Taiwan. As phytochemicals are evolved as part of the plant defense system in response to environmental stress, major phytochemical compositions between species may diverse and largely differ in their relative abundance [6, 7]. Indigenous plants in Taiwan, especially endemic species, are therefore precious sources of novel pharmacologically active compounds.

Modern chemical purification and identification technologies have allowed the identification of many novel phytochemicals from plants. In the past few decades, efforts have been made to explore novel phytochemicals from indigenous plants in Taiwan. A few of them were further examined for their biological activities, including cytotoxic activity against cancer cells, antiplatelet activity, and antituberculosis activity. With the accumulating information on the biological activities of these plant-derived compounds from indigenous plants in Taiwan, there is still no comprehensive database which links the novel chemical structures and their known pharmacological activities in a quantitative manner. Such information may not only improve our understanding to indigenous plants in Taiwan, but also facilitate the development of new therapeutic agents. An integrated platform with convenient search function for chemical structures and known biological activities is of great value. As a comparison, databases of Indonesian herbal constituents and aldose reductase inhibitors have been constructed and have enabled the building of quantitative-structure activity relationship (QSAR) models for virtual screening of novel aldose reductase inhibitors, potential therapeutic agents for diabetic retinopathy, and related neuropathy diseases [8].

 The presented database is designed to center with compounds identified from indigenous plants in Taiwan which have been examined for their anticancer, antiplatelet, or antituberculosis potential. The biological activities of a total of 99 indigenous species, including 29 endemic and 3 naturalized species, are curated into the database, with 5243 records of anticancer, antiplatelet, and antituberculosis activities, respectively. The curated data includes the recent isolated antitubercular flavonoids with promising minimal inhibitory concentration (MIC) [9, 10]. With the emerging prevalence of multidrug-resistant tuberculosis and lack of effective treatments [11, 12], these phytochemicals are especially valuable sources serving as promising candidates and/or QSAR skeletons for novel antituberculosis drugs development. Similar strategies are also applicable for anticancer and antiplatelet drug development. To our knowledge, this is the first publicly available database containing quantitative pharmacological data of phytochemicals from indigenous plants in Taiwan.

## 2. Construction and Content

The TIPdb database is implemented using MySQL Server Edition 5.1. The TIPdb website is publicly available at http://cwtung.kmu.edu.tw/tipdb. The web interface and all functions are implemented using PHP and JavaScript languages. The software libraries of jQuery UI [13] and Google Chart Tools [14] are utilized to make menus and sortable tables.

### 2.1. Database Content

**Figure 1:**
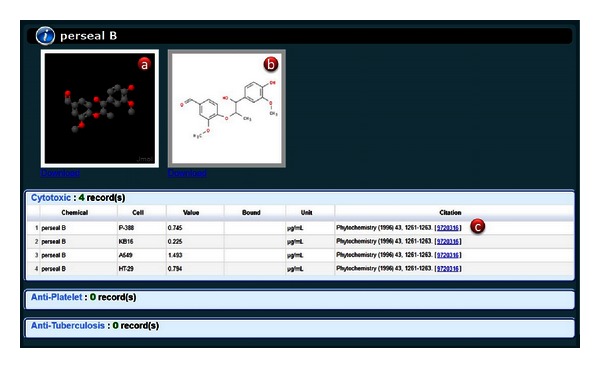
Content of a typical TIPdb entry. (a) 3D structure, (b) 2D structure, and (c) activity data and citation.

Indigenous plants and corresponding chemical compositions were extracted and manually curated from the published literature. Taxonomy classifications were manually assigned to the indigenous plants. Experimental information of cytotoxicity against cancer cells, antiplatelet activity, and antituberculosis activity of the chemical compounds was also manually curated from the published literatures (Figure 1).

For cytotoxicity data, the curated information includes cell type, assay condition (effective concentration (EC) or test concentration measured by MTT assay), activity value, and assay bound. For antiplatelet compounds, both chemical and inducer names with their concentrations are curated in addition to activity value. The information curated for antituberculosis compounds includes testing strain, activity value (MIC), and assay bound. Plants which were published in the literature with synonym names were manually changed to accepted names, following classification in the Flora of Taiwan, 2nd edition [5]. Such examples include *Persea obovatifolia *Kost.(now classified as *Machilus* genus) [15] and *Hernandia sonora* (now as *Hernandia nymphaeifolia*) [16]. 

Chemical structure files were either manually created or downloaded from PubChem database [17]. MySQL Server Edition 5.1 was utilized to implement databases. Web user interface and functions were implemented using PHP and JavaScript languages. The java applet-based program Jmol [18] has been extensively used in many websites for interactive displays of structure of biomolecules and chemicals. For example, Protein Data Bank (PDB) [19] and PupDB [20] utilize Jmol to display protein structures with ligands and pupylation sites, respectively. The chemical information resource ChemSpider also displays chemical structures using Jmol [21, 22]. In this study, TIPdb integrates Jmol for interactive displays of chemical structures.

## 3. Utility and Discussion

TIPdb is a database of phytochemicals from Formosan indigenous plants aiming to provide an easily accessible web service for data management, analysis, and novel therapeutic agent screening. Citation information is provided for accessing the original article source. For articles indexed in PubMed databases, hyperlinks to PubMed citations are available. 

The analysis of the bioactive chemical structures in TIPdb may provide insights into structure diversity and functional groups and substructures of the chemicals. For example, quantitative structure-activity relationship (QSAR) models can be developed based on the collected chemicals and activities. To facilitate such applications, several tools are constructed and integrated into TIPdb to provide useful functions of browsing, searching, and interactive displays of chemical structures. The functions of the integrated tools are introduced in what follows.

### 3.1. Browse Tool

**Figure 2:**
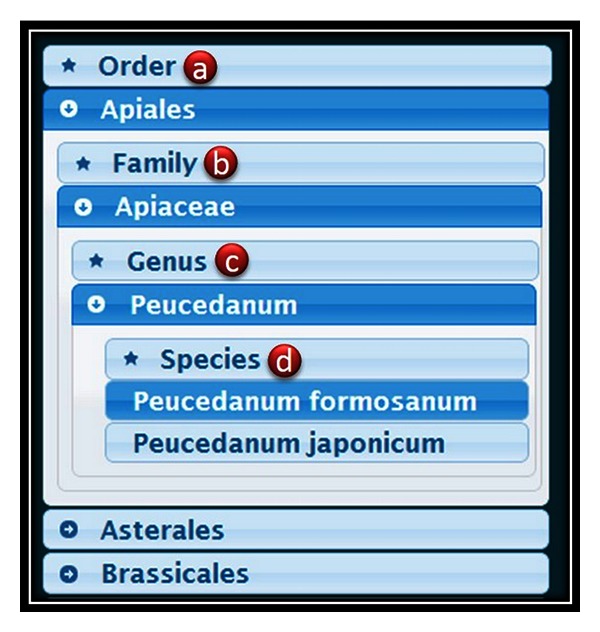
The taxonomy toolbar of browse tool. Users can browse plants by sequentially select (a) order, (b) family, (c) genus, and (d) species.

Users can browse TIPdb by selecting the “Browse” option. The left panel will show a taxonomy toolbar. The Taiwan indigenous plants in TIPdb are categorized into a taxonomy tree. Users can browse the taxonomy tree in the sequence of order, family, genus, and species (Figure 2). Corresponding chemical compounds from the plants will be shown in the right panel as a sortable table. By clicking the caption of a specific column in the sortable table, data of the selected column will be sorted in the output table. Users are allowed to specify the number of rows shown per page.

To browse details of a specific chemical compound, users can click the chemical name to show its corresponding activities of cytotoxicity against cancer cells, antiplatelet activity, and antituberculosis activity in the bottom panel. The browse results are presented as a sortable table. Chemical structures of the compounds are shown as an interactive program by using Jmol. 

### 3.2. Search Tools

**Figure 3:**
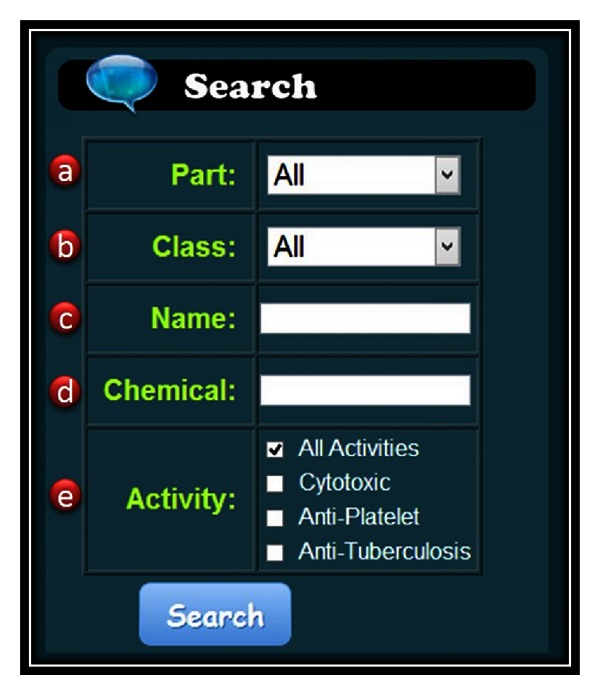
The search tools. Five search tools are available, including (a) the part of the plants, (b) the class of the chemicals, (c) botanical name, and (d) chemical activity.

For retrieving entries of interest, TIPdb provides a search tool using keywords of botanical name, part, chemical name, chemical class, and activity (Figure 3). The search fields can be individually or simultaneously utilized to filter entries. The research results will be shown in the right panel with links to detailed information of chemical structure and activity.

### 3.3. Interactive Tool for Protein Structure

TIPdb incorporates the Jmol applet of latest version 13.0 for interactive displays of chemical structures. Users can either use the user interface or scripting console to manipulate chemical structures. There is also a link for downloading chemical structure file.

## 4. Conclusions

Phytochemicals from indigenous plants in Taiwan could be potential drugs. A structured and searchable database, TIPdb, was constructed to serve as a comprehensive and standardized searching resource for anticancer, antiplatelet, and antituberculosis activities. The chemical structures of these phytochemicals are also curated in the database to provide a great opportunity to develop quantitative structure-activity relationship models for high-throughput screening of potential anticancer, antiplatelet, and antituberculosis drugs. The unique content of TIPdb is expected to be a useful resource for drug discovery.
